# A mixed-methods study exploring women’s perceptions and recommendations for a pregnancy app with monitoring tools

**DOI:** 10.1038/s41746-023-00792-0

**Published:** 2023-03-24

**Authors:** Natasa Lazarevic, Carol Pizzuti, Gillian Rosic, Céline Bœhm, Kathryn Williams, Corinne Caillaud

**Affiliations:** 1 Biomedical Informatics and Digital Health, School of Medical Sciences, Faculty of Medicine and Health, The University of Sydney, Sydney, NSW Australia; 2 Charles Perkins Centre, The University of Sydney, Sydney, NSW Australia; 3 Nepean Blue Mountains Family Metabolic Health Service, Department of Endocrinology, Nepean Hospital, Sydney, NSW Australia; 4 School of Physics, Faculty of Science, The University of Sydney, Sydney, NSW Australia

**Keywords:** Health services, Medical research

## Abstract

Digital health tools such as apps are being increasingly used by women to access pregnancy-related information. Conducted during the COVID-19 pandemic, this study investigated: (i) pregnant women’s current usage of digital health tools to self-monitor and (ii) their interest in theoretical pregnancy app features (a direct patient-to-healthcare-professional communication tool and a body measurement tool). Using a mixed methods approach, 108 pregnant women were surveyed and 15 currently or recently pregnant women were interviewed online. We found that pregnant women used digital health tools to mainly access pregnancy related information and less so to self-monitor. Most participants were interested and enthusiastic about a patient-to-healthcare-professional communication tool. About half of the survey participants (49%) felt comfortable using a body measurement tool to monitor their body parts and 80% of interview participants were interested in using the body measurement to track leg/ankle swelling. Participants also shared additional pregnancy app features that they thought would be beneficial such as a “Digital Wallet” and a desire for a holistic pregnancy app that allowed for more continuous and personalised care. This study highlights the gaps and needs of pregnant women and should inform all stakeholders designing pregnancy digital healthcare. This study offers a unique insight into the needs of pregnant women during a very particular and unique period in human history.

## Introduction

Maternal and neonatal disorders were among the top ten causes of global burden of disease in 2019^1^. Better access to perinatal healthcare would help to reduce preventable morbidity associated with pregnancy^2^. The increase in access to and use of smartphones presents a unique opportunity to transform and improve how women monitor their own health during pregnancy, especially for those living in remote regions. Furthermore, the COVID-19 pandemic has emphasized the need for more effective digital health interventions, better data collection, and continuity of care^3–5^.

Research demonstrates that pregnant women frequently search the internet and use digital health tools such as apps to access pregnancy-related information^6–8^. The usage of pregnancy apps either persisted or increased during the COVID-19 pandemic, partly due to the decreased access to in-person healthcare services^9^. However, several recent scoping reviews and cross-sectional studies have highlighted the need for better quality apps with greater content credibility^8,10–14^. Most pregnancy apps are primarily focused on providing educational information, and do not include self-monitoring features - even though there is evidence that these features can assist with behaviour changes that lead to improved healthcare outcomes^12,15^. For instance, Willcox et al. (2017)^16^ demonstrated that their mHealth digital health intervention, which required users to set goals and self-monitor, had regular user engagement that led to lower gestational weight gain and increased physical activity. Moreover, Dahl et al. (2018)^17^ showed that their Healthy Motivations for Moms-To-Be app that used behaviour change techniques such as goal setting was able to promote healthy eating behaviours.

Cross-sectional studies have suggested that pregnant women are eager for app features that connect them more directly to their chosen healthcare professionals^9,18^. As has been previously suggested, an app feature that allows healthcare professionals to remotely monitor their patients’ progress has the potential to make healthcare more continuous and accessible at a reduced cost^19,20^. A recent study tested the feasibility of remote monitoring and asked pregnant participants to monitor their weight, heart rate, blood pressure, physical activity and sleep patterns daily using a smartwatch, a blood pressure machine and a scale alongside attending 4 in-person clinic consults. They found that home monitoring with devices alongside in-person consults was feasible^21^. Thus, there is an opportunity to develop a digital health intervention that integrates tools that allow self-monitoring of multiple health parameters with remote monitoring by and communication with healthcare professionals.

When combined with other clinical parameters, monitoring body shape changes using anthropometry (e.g., measuring weight) during pregnancy can indicate the risk of developing certain health conditions such as gestational diabetes (GDM), obesity, pre-eclampsia, and the need for a caesarean delivery^22–25^. These potential risks are not isolated to the expectant mother, as they can result in neonatal premature birth, neonatal mortality, and early childhood obesity^26–28^. Historically, the most common anthropometric measurement used for perinatal health assessment is pre-pregnancy Body Mass Index (BMI). However, this measure does not capture the distribution and the percentage of a person’s body fat nor their genetic risk of disease. As such, two people with completely different body shapes and sizes can fall into the same BMI category yet have drastically different risk profiles for developing adverse health outcomes^29–33^.

Monitoring BMI as well as gestational weight gain (GWG) is now the standard approach to assess risk and provide recommendations to pregnant patients. However, other anthropometric and clinical parameters in addition to BMI and GWG are needed to help stratify disease risk as not all individuals are at equal risk of developing certain diseases such as GDM and preeclampsia^33,34^. For instance, a prediction tool for the early identification of GDM in pregnant women with obesity combined anthropometric measures with other measures such as blood pressure^35^.

In addition to this, studies reporting the reliability and accuracy by which doctors take anthropometric measurements revealed that while BMI is reliably measured, measures of hip circumference, waist circumference, and waist-to-hip ratio are measured less reliably especially in patients with obesity^36,37^. Considering this, novel digital approaches to taking body measurements should be explored (1) to allow for more consistent and convenient measurements for patients and (2) to support patients self-monitor these anthropometric measures that are generally difficult and inconvenient to take themselves using a measuring tape.

The overarching aim of this study was to evaluate pregnant women’s interest and willingness to use digital health monitoring tools. To help gauge their attitudes more tangibly, we asked them about a hypothetical app, with a body measurement self-monitoring tool that extracts digital measurements of the body from photos taken by users on their smartphones, and a patient-to-healthcare professional communication tool that allows for direct communication with user’s healthcare professionals. Thus, using a combination of online surveys and interviews, this study investigated: (i) pregnant women’s current usage of digital health tools to self-monitor, and (ii) their interest in the hypothetical pregnancy app.

## Results

### Demographic data

The demographic characteristics of the 108 survey participants (101 completed and 7 partial responses) are presented in Table 1. Three quarters of the participants (76%) were in Australia. The majority of the participants were between the ages of 18 and 35 (71%), were in their second or third trimesters (84%), were experiencing their first pregnancy (55%), were in the normal and overweight BMI ranges (71%), completed tertiary education (71%), and reported no health conditions (65%) (See Supplementary Table 1 for more demographic details). The majority of the interview participants were recently pregnant and in their postnatal period (10/15, 67%), while the rest were pregnant at the time of the interview. Fifty-three percent (8/15) reported that it was their first pregnancy. Majority of participants were in Australia (10/15) (Supplementary Table 2).

**Table 1:** Demographics of the total of 108 survey respondents.

	*n*	%
Continent		
Australia	82	75.9
North America	8	7.4
Africa	7	6.5
Europe	7	6.5
Asia	4	3.7
Age		
18–34	76	71.0
35–50	31	30.0
Trimesters		
First trimester (1–13 weeks)	17	15.7
Second trimester (14–27 weeks)	48	44.4
Third trimester (28–42 weeks)	43	39.8
Gravidity		
None	59	54.6
One	36	33.3
More than one	13	12.0
BMI		
Underweight	7	6.5
Normal/Healthy	47	43.5
Overweight	29	26.9
Obesity	25	23.1
Education		
Tertiary education	77	71.3
Secondary education	21	19.4
Vocational qualification	9	8.3
No formal education	1	0.9
Reported health condition		
Yes	38	35.9
No	69	65.1

The health conditions respondents selected from include: Type 1 and 2 diabetes, hypertension, depression, Polycystic Ovarian Syndrome, non-alcoholic fatty liver disease, obstructive sleep apnoea and other. Ancestry was entered by respondents as free text and grouped into regions.

### Outcomes

**Fig. 1:**
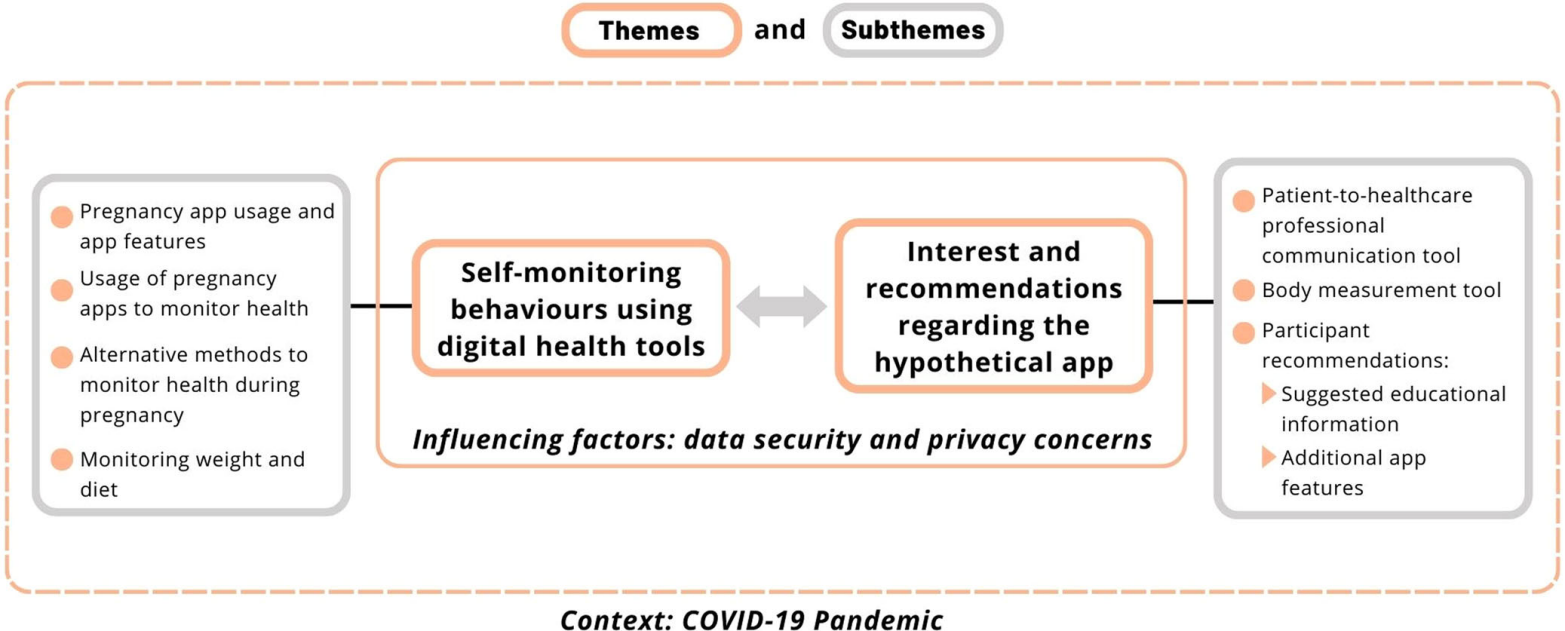
Themes and subthemes arising from the interviews. Themes: orange boxes. Subthemes: grey boxes. The relationship between the themes is depicted using a grey arrow. The dashed orange box outlines the context, COVID-19 pandemic.

Two main themes arose from the interviews: (i) Self-monitoring behaviours using digital health tools, and (ii) interest and recommendations regarding the hypothetical app. The survey and interview results are described within these themes. It must be noted that this study was conducted during the COVID-19 pandemic, and this context is relevant to the study findings (Fig. 1). In fact, the pandemic overall influenced how willing participants were to use digital health. Of the interview participants, 73% (11/15) agreed that the pandemic influenced how willing they were to use digital health tools or access information online: *“But yes, definitely having COVID as a restriction. Yeah, far more inclined to use a digital tool”* [IP 1].

### Self-monitoring behaviours using digital health tools

Most survey participants (72%) reported that they used pregnancy apps during their pregnancy. As shown in Table 2, the apps were primarily used as a source of information (65%), for education (42%), for self-monitoring (45%), and for reassurance that the pregnancy is going well (36%). Similarly, 67% (10/15) of interview participants stated that they used pregnancy apps (See Supplementary Table 3 for a breakdown of which apps were used). While one of the most common features of pregnancy apps is baby development information and baby size-to-fruit-size comparison, only 4 people used it for that purpose.

**Table 2:** Survey participant self-monitoring behaviours.

	*n*	%
I feel that I can monitor my pregnancy easily from home		
Strongly Agree	5	4.6
Agree	47	43.5
Do not agree or disagree	23	21.3
Disagree	28	25.9
Strongly disagree	5	4.6
Do you use any activity trackers such as fitbits or smart watches during pregnancy?		
I use activity trackers	41	38.0
I do not used activity trackers	67	62.0
For what purposes do you use pregnancy apps?		
As a source of information	70	64.8
For education	45	41.7
For self-monitoring	49	45.4
For reassurance that the pregnancy is going well	39	36.1
Other	8	7.4

Of those interview participants that didn’t use pregnancy apps, three explained that they felt they did not need to use it because it wasn’t their first pregnancy, and they knew what to expect. All the interview participants that used pregnancy apps emphasized that the app user experience, interface, and customisability were important factors when deciding which apps to use: *“I’m very impatient when it comes to user interfaces, so if something doesn’t work easily for me. I’m gone”* [Interview Participant (IP) 7]. In this regard, participants mentioned that there was a lack of personalization in apps, especially for symptom tracking. For instance: *“…I mean, sometimes it made me less reassured…For example, when I felt so awful, and that carried on into my third trimester and all the apps were telling that I was going to start to feel better…but because it sort of contradicted what the health professionals were saying”* [IP 4].

Regarding the utilisation of pregnancy apps to monitor health, we found that while most participants used digital health resources to access pregnancy-related information, only 45% (49/108) of survey participants and 30% (5/15) of the interview participants reported using an app to monitor their health during pregnancy. Also, when survey participants were asked whether they agreed or disagreed with the statement “I feel that I can monitor my pregnancy easily from home”, only 44% agreed. One of the more common reasons why interview participants reported not using an app to self-monitor their health during pregnancy was because they had been pregnant before and knew what to expect. However, interview participants who tracked a health condition during pregnancy tended to monitor their health more frequently, *“…I have chronic hypertension. And I actually just wound up using a spreadsheet to track that”* [IP 3]. Among the interview participants who used an app to monitor their health during pregnancy, four stated that the app chosen was not designed for pregnancy because they could not find pregnancy specific versions. These included Kegel exercise, diet, and calorie counting apps, *“…All of the like health tracking…none of it has support for pregnancy, which is very annoying”* [IP 3].

However, apart from using digital health tools, some women had reported that they have their own approaches to self-monitoring, such as note taking, their personal memory, photos, and excel spreadsheets. Participants used such self-monitoring methods to track health data such as, their blood pressure, blood glucose insulin, calorie intake, medications, physical activity, and symptoms. Another method of recording health data included activity trackers, which only 38% of survey participants reported using during pregnancy. Three quarters of those participants reported that they achieved their exercise goals more than once weekly using their activity trackers (Table 2).

### Self-monitoring behaviours using digital health tools – monitoring and data security and privacy concerns

The interview participants that used apps to self-monitor weight or diet said that they tracked their weight *only* when prompted by a notification to record their weight. One remarked that when weight was tracked, no feedback was provided, “*And I did track my weight in it. But I mean, that was really just a place to put it, it didn’t really provide any feedback or anything”* [IP 7]. Notably, only 4% (4/108) of survey participants reported using any app to monitor their diet during pregnancy. Another factor affecting willingness to use digital health tools was not related to feeling that they could monitor themselves from home, but concerns about data privacy and security. 63% of the survey participants reported having concerns about data privacy and security in relation to using the pregnancy apps. Moreover, the multiple logistic regression of survey responses found that, holding all other predictor variables constant, having “no concerns about data privacy and security issues in pregnancy apps” (*p* < 0.0001) was a significant predictor for the likelihood that survey participants used pregnancy apps (95% CI 2.81,7.58: odds ratio = 127.84). BMI, age, gravidity, “feel that they can monitor themselves from home”, “use digital health more now that they are pregnant” and “reported a health condition” were non-significant predictors (Table 3). Some interview participants mentioned that they had surpassed their privacy concerns during the pandemic because of the unorthodox situations they had to face and because of their need for care and remote monitoring by healthcare professionals: *“I worked with a lactation consultant when I got home from the hospital. And we had to do virtual appointments because of the pandemic. And that meant that I literally texted her on WhatsApp, like pictures of naked pictures of my boobs, which, you know, is not something that I would have opted to do if there hadn’t been a pandemic and I could have seen her in person…But because of the pandemic, I was like, “Great, I hope this doesn’t get hacked and leaked somewhere””* [IP15].

**Table 3:** Results of statistical analyses using logistic regression to determine likelihood of pregnancy app usage.

Predictor variables	*P* value	Estimate	Odds ratio	95% Confidence Interval
Likelihood of pregnancy app usage (response variable) Pseudo R^2^ (0.482) and *β* (−3.631)				
Concerns about data privacy and security issues [No]	<0.0001	4.85	127.84	2.81, 7.58
Concerns about data privacy and security issues [Yes]	0.143	1.58	4.86	−0.40, 3.97
Use digital health more now that they are pregnant [Yes]	0.130	1.24	3.46	−0.40, 2.89
Health condition [yes]	0.139	1.34	3.80	−0.32, 3.30
Feel that they can monitor themselves from home [Strongly Agree]	0.370	−1.55	0.21	−5.00, 2.06
Feel that they can monitor themselves from home [Agree]	0.870	−0.16	0.85	−2.16, 1.76
Feel that they can monitor themselves from home [Disagree]	0.483	0.866	2.37	−1.52, 3.40
Feel that they can monitor themselves from home [Strongly Disagree]	0.490	1.22	3.38	−2.00, 5.29
BMI [Obesity]	0.779	0.26	1.30	−1.54, 2.20
BMI [Overweight]	0.318	1.05	2.86	−0.89, 3.32
BMI [Underweight]	0.369	−1.28	0.28	−4.29, 1.42
Age [35–50]	0.251	0.97	2.64	−0.61, 2.79
Gravidity	0.549	−0.30	0.74	−1.21, 0.80

Odds ratios and Confidence Intervals are reported for each test.

### Interest and recommendations regarding the hypothetical app - patient-to-healthcare professional communication tool

**Fig. 2:**
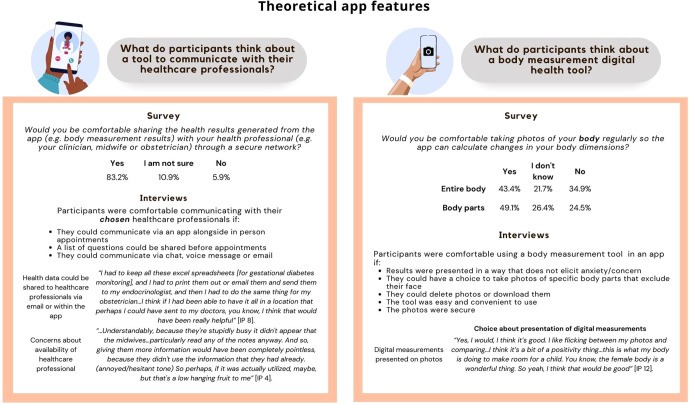
Participant thoughts about 2 theoretical app features. The figure summarises both the survey and interview participant responses when asked about a tool to communicate with their healthcare professionals and the body measurement digital health tool.

All survey and interview participants were asked how comfortable they would be to use a patient-to-healthcare professional communication tool (Fig. 2). The vast majority of survey participants (83%) expressed that they would be comfortable sharing the health results generated from the theoretical app with their healthcare professional (e.g., their clinician, midwife or obstetrician) through a secure network. Complementing the survey findings, all the interview participants (100%, 15/15) shared that they would be happy to communicate via an app with their *chosen* healthcare professional/s: *“Oh, of course, I’d love that…It would make it a lot more accessible. But as long as I know that she [their healthcare professional] is the one whom I’m going to be seeing even in my next appointment…That is valuable, you know, that sort of established care, continuous care with one person like that one point of contact.”* [IP 11]. In addition to this, participants wished not only to communicate with their healthcare professionals, but also share relevant health data. Participants mentioned that they would communicate with their healthcare professional via an app because communication via the phone or email was not possible or feasible, *“…Because sometimes you just have the simple question, and, you know, having to wait for an appointment and go through everything is just harder*.” [IP 2]. The form of communication within an app that participants said they would prefer were communicating via chat, voice message, or email. For example, a participant shared their experiences communicating with their strength coach via voice message: *“Just being able to send her a voice message or a thing on the other communication app we used and then she’d get back to me when it was convenient for her without, you know, having for me to wake up at a certain time…I guess asynchronous communication. And I found that quite helpful”* [IP 9]. In line with the participants’ willingness for asynchronous communication, a common app feature participants mentioned they would find helpful was sending the questions they plan to ask their healthcare professional *before* their upcoming appointment.

Another point participants raised was how digital health could bridge the communication not only between patients and health professionals, but also between specialists themselves. Several participants reported the difficulties they encountered during pregnancy when they needed to communicate with multiple healthcare professionals or specialists. One participant explained, for instance, how difficult it was to remember what their obstetrician wanted them to ask their endocrinologist and suggested how the communication between specialists could be bridged by digital health, “*…My obstetrician is a bit old school, but he would often use a voice recorder and record his notes…Maybe something that could just capture a couple of points and then I could play that back to the endocrinologist or vice versa…I think that would be really useful.”* [IP 8].

### Interest and recommendations regarding the hypothetical app - body measurement tool

All participants were also asked how comfortable they would be to use the body measurement tool described in the study design section of the methods (Fig. 2).

About half of the survey participants were willing to take photos of their entire body (43%) and/or body parts (49%) so the app could automatically measure their body changes over time. The majority of people who were willing to take photos of their body parts were also willing to take photos of their entire body (43/52). In line with the survey results, 33% (5/15) of interview participants immediately agreed that they would be comfortable taking photos of themselves and using a body measurement tool, as it would help them monitor their health, quantify changes, and store a collection of pregnancy photos. Most of the interview participants (12/15) were interested in using the body measurement tool to track leg/ankle swelling: *“My feet, they started swelling. So, I was taking pictures of them to compare like, okay, is it swollen from last week or this week, but that was towards the end. So, I would use that feature, like to track the swelling on my feet*” [IP 2].

Notably, a larger proportion of interview participants (47%, 7/15) expressed that they would use a body measurement tool to measure specific body parts only. These participants were hesitant about taking photos that would include their faces due to privacy and body image concerns: *“Because you mentioned ankles, I think I would be totally okay, taking pictures of my ankles like that…But in my mind, I was thinking about bumps or my face or something that feels a bit different somehow…faces are obviously identifiable, and then bumps are not identifiable in the same way, but it feels a bit more personal than taking a photo of my ankles or fingers or something else. So yeah, I think that would definitely play into it. What I was taking the photo of for sure*” [IP 10]. The privacy concerns were not isolated to the inclusion of their faces in photos, but to concerns about, “*Who keeps that information*?” [IP 1]. When the interviewer explained that only the digital measurements extracted from their photos would be stored and used to train a machine learning system, and that they would choose who their data was shared with (family/friends/healthcare professional), participants reported that their concerns were alleviated. Similarly, 68% of survey participants responded “yes” to being comfortable with the use of their anonymised data [digital measurements extracted from photos] to train and develop the app technology while using the app (21% = I am not sure and 11% = No). In addition, survey participants were asked how interested they were to use an app that learns from their anonymous data (i.e., machine learning) to assist in the identification, prediction, and prevention of adverse health outcomes during pregnancy and there was moderate-high interest (76%) in using such an app. The interview participants that were not comfortable using a digital body measurement tool (20%, 3/15) had both privacy and body image concerns, “*I think that would make me very uncomfortable…And I’d be worried about my privacy too*” [IP 4]. In fact, when survey participants who responded “no” (28/106) to the question of being comfortable taking photos of their body parts regularly to extract digital measurements were asked to elaborate why, their explanations included discomfort/uneasiness, privacy/security concerns, body image/mental health issues, and credibility. In line with participants’ concerns around body image issues, one participant with a nutritionist background voiced their concerns about the lack of diversity of current anthropometric standards/guidelines, *“What is typical? How do you come up with references or standards, when there’s this level of heterogeneity in body shapes and body types? And just in general, who stores fat where, you know, so for all you know its fluid accumulation, it’s fat, or they have goiter?*” [IP 11] and, consequently, on the potential unindented yet negative consequences of the use of monitoring tools on pregnant women’s mental health.

### Interest and recommendations regarding the hypothetical app - suggested educational information

Interview participants were given the opportunity to make additional comments regarding the use of digital health tools to monitor pregnancy. In response, participants shared which educational information (Table 4) and additional features (Table 5) they would include in an app or digital health tool. The following are a selection of the issues they experienced during pregnancy and some of their ideas for potential solutions. All the 15 interview participants mentioned that the educational information provided in apps or digital health tools could be improved. Some participants noted that more support and information should be provided about the initial stages of pregnancy, before their first pregnancy confirmation appointment at 8–9 weeks, and before their 20-week morphology scan. One participant explained the anxiety that they felt before their first pregnancy confirmation appointment, and how other users in a pregnancy app they used (Peanut app) felt the same need of support and reassurance. Four participants added how challenging it was to access guidelines that were pregnancy specific and that there is a need for credible information to help interpret health results. Aside from access to more pregnancy specific guidelines, there was a desire to learn more about how their bodies change during pregnancy outside of how their baby is developing.

**Table 4:** Educational information for a pregnancy app or other digital health solution.

What types of educational information do interview participants want?	Participant quotes
Support and information about initial pregnancy stages before the first doctor’s appointment (at 8-9 weeks) and before the 20-week morphology scan	*“A lot of us on the app [Peanut], were very worried, like, you know, who had confirmed pregnancies only based on the home kit, but who had to wait till the eighth or the ninth week… And it was just such a real experience for me because, you know, you don’t know what it is like till the time you actually go for your first ultrasound…So I think some sort of support in the very initial phases that will be useful to incorporate in a pregnancy app. (nervous tone throughout)”* [IP 11].
Not only information about fetal development (and fruit comparison), but information about how the female body changes during pregnancy	*“…And I think what would have been great was to understand how my body’s changing, how my hips are spreading apart, the strains putting on my muscles, and then things I can do to help with that, to help manage that pain…Even then having like a digital tool to help you understand like, you know, there’s loads of things that help you visualize how your baby’s developing, but there’s less about how your body’s changing, and then what you can do to help you understand and help you manage that would be super helpful”* [IP 13].
Pregnancy specific guidelines within the app to help interpret tests such as blood tests and risk factors (related to pre-pregnancy BMI)	*“I think there’s still a space for a really good pregnancy tracking app that shows, you know, all of the common things that they’re looking for you. You take millions of blood tests, and they give you back like all of these, like, random numbers that you spend a bajillion years trying to figure out what they mean…My chart [the app] is the thing that most people use. They’re all calibrated for non-pregnant people. And so, the app will come back and tell you that you have, you know, you have an elevated white cell blood count. And it’s like, who cares all pregnant women have an elevated white blood cell count, which is only after a bunch of googling…And so there’s this situation where the medical system is giving you this data, but there’s nothing useful to interpret it at all (frustrated tone). And the tools that they give you for interpretation often aren’t geared towards pregnancy. And so having an app that would show me how things were progressing on all of these things that they were tracking…It would have been really, really nice”* [IP 3].
Breast feeding - information about diverse experiences and the top 10 common complaints or problems postpartum	*“…honestly, for me, the biggest information gap came with breastfeeding. Um, and I definitely, I felt like a complete absence of support, especially from health care providers in that regard. And so, if there had been resources related to breastfeeding, that were a little bit beyond just sort of, like, you know, your baby should latch. I don’t know, it was just terrible”* [Participant IP 15].

The table summarises the points interview participants raised regarding the educational information that they would like to be included in a pregnancy app or other digital health solution. The Table includes selected interview participant quotes related to some of the points raised.

**Table 5:** Additional features for a pregnancy app or other digital health solutions.

What types of features did participants want an app or digital tool to include?	Participant quotes
Export/share tool of app data and educational information	*“Yes, but the user interface would have to be very sensitive. So, the app that I have doesn’t allow screenshots at all for privacy reasons. And that was frustrating because sometimes I was talking to my mom or my friend who had preeclampsia. And I was like, here are the numbers they got for me today on my protein test. And it would be really weird to have a button under my urine protein test that says share with a friend. And yet, that’s exactly the feature I wanted. So, you know, something that said, like export might have been a little more appropriate”* [IP 3].
Connect users to research publication database	*“I found the app that I used, like they had a bit of information, but it was super basic and general, which of course, that’s what you would expect in the app. But if they actually had links to solid research, and a database where you could go and look at that stuff. I think that would be really good”* [IP7].
Shared user interface with healthcare professionals	*“So particularly with the preeclampsia, I had to report the data back to my doctor. And so, it was a little kludgie, the medical apps, they just have like this text box for you to fill stuff in…And I would come in, and I would have my own graphs…And so, I would have my own graphs. And I would, like, bring them printed out so that we could talk about them. And it would have been so nice if there was some sort of like shared interface that we could go over the data together with and be like, here’s, you know, here’s what we’re seeing”* [IP 3].
'Digital Wallet’ that includes digital copies of resources provided by hospitals, pregnancy antenatal cards, receipts, scripts etc.	*“I would much prefer to have gotten everything digitally…Even scripts…I just never really understood why I had to keep it with me, why couldn’t they just email it to each other, it’s just like, on my file. So that kind of thing I felt was really weird. And, you know, obviously very old systems”* [IP 13]. *“I do think that having a digital wallet would have been literally life changing. That would be amazing, something secure, that you could store all of that documentation in”* [IP 7].
Symptom tracker - could work like a contraction/ kick counter	*“I just really would have loved to have something to monitor how much I was being sick… I don’t know even if you just like you know, push a button every time you were sick, and then it could calculate over a period of time how often it would be happening…Well, the app, I had actually had a kick count. So, like, you could press it every time you felt kicks and then it would track that…So yeah, like something similar to that for the vomiting would have probably really helped me because I’d go into the hospital, and they would be like*”*, “How many times have you been sick in the last 24* *h?*” *I’d be like, “I don’t know. 1000? Like, I fill buckets? I don’t know”* [IP 5].
Gestational diabetes monitoring	*“Maybe something about the gestational diabetes, because I think that’s quite common. You know, a lot of women, you know, particularly older women sort of have that issue…I think would have been really useful”* [IP 8].
A holistic app (from antenatal to postpartum)	*“…There’s just so many different apps for so many different things, it would be good to have something that kind of combines everything into one, especially now that I’m looking towards, like downloading apps for sleep for the baby to track their sleep, there’s breastfeeding apps to track how much milk they’re drinking…So, like I’m just finding that my phone is getting filled with all these different apps. And you have to keep inputting your information into every app, you know, if it’s about me, it’s my age, my height, my weight, how far along I’m in my pregnancy and things like that. And if it’s about the baby, you have to input all that information. So, if there was some sort of app that could follow you from pre pregnancy right through to baby being born, I think that would be quite beneficial”* [IP 12].

The table summarises the points interview participants raised regarding the app features that they would like to be included in a pregnancy app or other digital health solution. The Table includes selected interview participant quotes related to some of the features raised.

Despite these limitations, many participants felt that the support and information they received during pregnancy was still satisfactory. They reported though that postpartum support was inadequate - such as access to more diverse breast-feeding resources - and that better communication about potential postpartum complications should be encouraged.

### Interest and recommendations regarding the hypothetical app - additional app features

With regard to possible app features, many participants stated that they would find a “Digital Wallet” that stores copies of their medical data (such as resources provided by hospitals, pregnancy antenatal cards, receipts, scripts, and referrals) extremely helpful. One of the major factors that annoyed participants was that they had to carry physical scripts or referrals to appointments. Another additional feature many participants mentioned was a tool to track their symptoms during pregnancy. A participant who experienced severe morning sickness or hyperemesis gravidarum during pregnancy mentioned how difficult it was to track the number of times they vomited. In particular, two participants expressed that there is a current lack of support for pregnant women who are diagnosed with gestational diabetes and suggested that a gestational diabetes tracking tool should be developed.

Several participants suggested that they would find a personalised list of appointments and appointment reminders useful because their busy schedule (due to their work and childcare responsibilities) makes it difficult to track their health appointments, as well as information about what to expect during those appointments.

In terms of data sharing, participants indicated that they would not only like to share their data with their healthcare professionals, but with their family and friends using an export or share tool.

To summarize, all interview participants besides one felt that there was room for an app that provided personalised support, tracked pregnancy at all stages from preconception to postpartum, and provided integrated and continuous care, in other words a more ‘holistic’ pregnancy app.

## Discussion

This study sought to investigate (i) pregnant women’s current usage of digital health tools to self-monitor, and (ii) their interest regarding two theoretical pregnancy app features. Using our mixed methods study design, we found that the majority of participants already used pregnancy apps and other digital health tools, but most did not use them to self-monitor. We found that participants primarily used pregnancy apps to access pregnancy-related information and receive updates about their baby’s development – which is consistent with previous cross-sectional studies^18,38^ – but many also expressed their desire for apps to also provide information about the changes occurring to their own bodies.

The majority of participants were interested and enthusiastic about a patient-to-healthcare-professional communication tool. While less than half of survey participants (43%) were comfortable taking photos of their bodies for the app, most of the interview participants (80%) were interested in using the body measurement tool to track leg/ankle swelling. Additionally, participants consistently raised the need for additional educational information and app/digital tool features that allow for more personalised and holistic care. These findings contribute to the growing literature on the needs and preferences of pregnant women during the COVID-19 pandemic^9,39^.

However, what are the barriers to using digital health to self-monitor? Based on both the survey responses and interviews, the largest barrier for using digital health to self-monitor was that the available tools did not meet the consumer demands. This is illustrated by our finding that only 51% of survey participants felt that they could monitor themselves from home and that interview participants often relied on other approaches to self-monitor such as using their memory or an excel spreadsheet.

In contrast to previous findings^9,18,40^, a significant barrier to using digital health found in this study was security and privacy concerns as well as concerns about information credibility and quality. This shift in concerns is likely due to: (1) the increasing spread of misinformation online, which participants mentioned as being the driver for their preference for their healthcare professional to be their main point of contact, and (2) poor data privacy, sharing, and security standards in pregnancy apps being widespread^41^. Thus, the quality of information and data privacy of pregnancy apps should be more formally assessed. Perhaps apps could be reviewed by a panel of experts and scored for information quality and privacy before they are uploaded to app stores, or a central app rating platform could be created^42^.

**Fig. 3:**
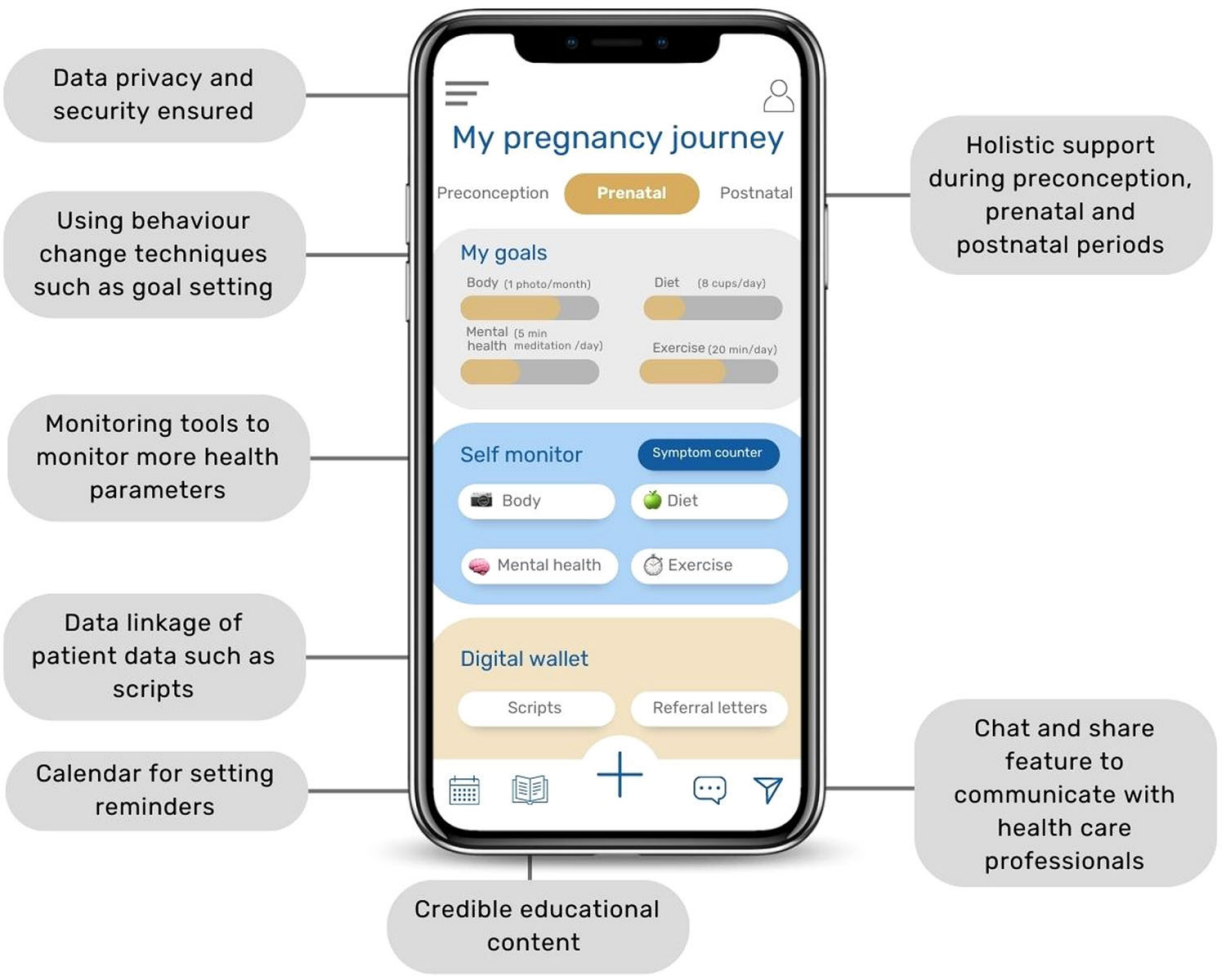
User-interface mock-up of pregnancy app features. The figure outlines which app features (grey boxes) pregnant women outlined that they would like an ideal pregnancy app to include.

Based on our study findings, the features pregnant women wanted to include in an ideal app can be outlined. When we asked about the theoretical app features of a patient-healthcare provider communication tool and a digital body measurement tool, interview participants were enthusiastic to share their views and even shared their thoughts about what an ideal pregnancy app should include. As a cohort, participants outlined that an ideal pregnancy app should: (1) provide holistic care for preconception, prenatal and postnatal support, (2) include credible information developed by experts/clinicians, (3) ensure data privacy and security, (4) include a patient-healthcare provider communication tool, (5) include a “Digital Wallet” with their patient data, (6) include a body measurement tool that measures body regions such as the ankle/foot region, (7) include monitoring tools for other health parameters such as diet, physical activity and mental health, and (8) include behaviour change techniques, such as reminders, goal setting, and providing personalised feedback on progress towards goals. A user-interface mock-up of these app requirements is depicted in Fig. 3.

Several pregnancy app reviews have shown that most commercial pregnancy apps include low numbers of behaviour change techniques especially for providing personalised feedback, goal setting and planning^11,43,44^. Additionally, pregnant women mentioned that a pregnancy app should have a well-designed user-experience and user-interface, though as demonstrated by numerous pregnancy app reviews the majority of currently available commercial pregnancy apps perform highly on usability^10,45–48^.

However, there are several benefits and barriers that would need to be considered when implementing the ideal app. The first benefit is that the body measurement tool within this ideal app could allow for consistent and convenient monitoring of body shape changes over time and allow for the collection of more diverse and complete data about body changes during pregnancy. With sufficient data, machine learning could help mitigate and stratify risk (i.e., your ankle swelling has not decreased for x days, please contact your healthcare provider). It is also promising that most participants were open to their anonymised data being used to train the machine learning system of a theoretical app. And that patient image-based assessment is becoming more widely accepted, especially for wound-care management^49^. However, an associated barrier of the body measurement tool and the other monitoring tools mentioned (such as for diet and physical activity tracking) is that with the introduction of any new health assessment measures or digital screening tools, there are concerns regarding overinterpretation of results and inappropriate linkage to disease risk. Results and feedback presented to users could also induce anxiety or lead to unnecessary clinical consults. As described by Capurro et al.^50^, digital screening tools can lead to overdiagnosis and potential harm to patients, and for this reason should be validated. Another factor to consider is the privacy of machine learning models and how that can be minimised by using anonymisation and data minimization tools^51^. However, the need to tailor body measures for ethnicity and cultural appropriateness is a challenge, which is evident as there are still no concrete and accepted BMI guidelines for different ethnicities. Thus, the clinical utility of health monitoring tools such as the described body measurement tool should be assessed by healthcare professionals and its accuracy should be validated in an iterative process.

The second benefit is that the patient-to-healthcare-professional communication tool embedded in the ideal app could be integrated within current healthcare systems. Integration of such tools has the potential to allow for asynchronous communication, remote monitoring, and continuous care. For instance, a mobile-based telehealth service in rural Bangladesh that allowed for remote consultation for maternal, neonatal, and infant health or emergencies was able to provide users with advice, preliminary diagnosis, reassurance, referrals, and scripts and promote healthy behaviours, such as regular healthcare consults^19^. A barrier to the implementation of such digital support apps is the required integration within current healthcare systems and workflows, which is notoriously challenging. However, studies have suggested that embedding technologies within existing systems is the preference^20^.

Lastly, all the participants emphasized their preference for a tool that enables communication with their *chosen* healthcare professionals. This is consistent with other studies that have demonstrated patients desire for more continuous care from their healthcare professionals during pregnancy^9,18^. However, for such interventions to be successful, the involvement of healthcare professionals is a must. A mixed methods study of healthcare providers during COVID-19 revealed that when telehealth consults replaced in-person care, the quality of care was impacted^52^. Other challenges mentioned in this study were the lack of digital literacy, patient monitoring, and disruption of patient-healthcare professional bonding. Thus, digital communication should not disrupt any in-person care and should be used in conjunction^39^. The type of communication, feedback, and time commitment provided to patients by healthcare professionals should also be carefully considered and accounted for in staffing profiles.

Thus, digital health interventions such as the described hypothetical pregnancy app in this study could enhance care in rural settings and/or support the flexibility of healthcare delivery especially during pandemics, where face-to-face contact needs to be minimised. Such a digital health service could employ a range of features that drive user engagement, encourage self-monitoring, communication with healthcare professionals, and ultimately lead to behaviour change.

This study has several limitations. Firstly, though the survey was tested for face and construct validity between authors and several external researchers, it was not pilot tested with pregnant women before it was launched. As outlined by several studies, it is recommended to test surveys for their validity and relevance with a pool of intended respondents^53,54^. However, as raised by Goodyear-Smith et al. (2015)^55^ and experienced by the authors, there are some challenges with pre-defining participatory and co-design approaches to involve research participants for human research ethics committee approval as the process can be iterative and unpredictable.

Secondly, we found that interview participants had several follow-up questions when deciding how willing they were to use the theoretical app features. Perhaps the format of using surveys to assess willingness to use theoretical features may not have been as appropriate of a method as the interviews. Participants may not have fully been able to understand the theoretical features from the short descriptions about them provided in the survey, and this may have impacted their responses. For instance, a relatively large proportion of survey participants responded, “I don’t know” when asked about their willingness to use the body measurement tool to take photos of their entire body (22%) and body parts (26%). Additionally, interviews can be used to not only assess the needs of users but using human-centred design approaches, prototypes can be developed with users^56^. After such a design process, future studies may consider displaying digital prototypes of theoretical app features created with users alongside questions related to it to provide survey responders more context.

Thirdly, a larger proportion of both survey and interview participants were highly educated (tertiary education: 71% and 53%), which could explain their hyperawareness about app privacy issues. This elicits the question of how transferrable the study findings are to more diverse groups. In addition, due to the random sampling method used for recruitment, representation across all demographics could not be achieved (such as for country location, ethnicity, BMI category, and socioeconomic status). This highlights the importance of designing accessible and culturally appropriate digital health solutions, which are reliant on the involvement of more diverse groups or several co-design iterations with different subgroups. Innovators should consider abiding by frameworks that promote digital health equity during development to prevent disparities in access to these tools^57,58^.

Overall, our findings demonstrate that pregnant women feel that there is a gap for a better pregnancy app or tool that allows for more holistic care from the prenatal to postnatal period and that could be integrated within healthcare systems. Their enthusiasm for a patient-to-healthcare-professional communication tool and interest in using the body measurement tool to track leg/ankle swelling illustrates that pregnant women are willing to use self-monitoring tools as long as they are accompanied by remote monitoring or connection with their healthcare professionals. Based on the findings of this study, researchers, innovators, and developers seeking to improve digital health services for pregnant women should consider incorporating the app features raised by pregnant women. We examined self-monitoring behaviours and recommendations for a hypothetical pregnancy apps during a very particular and unique period in human history. The question remains whether attitudes expressed here will continue in post-pandemic times. However, it is clear that such digital strategies are likely to improve the flexibility of healthcare systems to respond to such unpredictable events into the future.

## Methods

### Study design

A convergent mixed method approach was used. Quantitative and qualitative data were collected concurrently and analysed separately through surveys and interviews. The participants could complete both the survey and interview, but their data were not linked. The study findings and interpretations were triangulated from the combined data. At the end of both the surveys and interviews, participants were asked to share their thoughts about two theoretical app features: (1) a digital tool that would allow them to communicate with their chosen healthcare professionals, and (2) a body measurement tool, that extracts digital measurements of their body from photos taken on their smartphone. Specifically, participants were asked if they would be comfortable taking photos of their entire body or body parts regularly in an app that monitors how their body is changing and would give feedback about those changes. If participants wanted further elaboration, examples were provided of how the measurement tool could be used (such as, using the tool to monitor whether swelling in the ankle region was increasing or decreasing). Participants were then asked if they would feel comfortable communicating with and sharing health information with their chosen healthcare professionals.

### Ethics approval

Ethics approval was obtained from Nepean Blue Mountains Local Health District Human Research Ethics Committee, Australia (Ethics approval number: ETH00580). Online consent was obtained from all participants.

### Recruitment

Participants were eligible to complete the survey only if they were (1) currently pregnant, (2) able to provide consent, and (3) English literate. Participants were eligible for the interviews only if they met the criteria described above and if they were recently pregnant in the last 12 months. Eligibility was assessed for interview and survey participants upon completion of an online form. Participants were recruited both in person and online. In person recruitment was conducted at the Nepean Hospital Antenatal Clinic, Australia. Online recruitment was conducted via social media posts and advertisements as well as via email newsletter advertisements. All participants had the option to provide their contact details at the end of the survey if they wished to express their interest in participating in the interviews. Survey recruitment occurred during November 2020–May 2022 and the interviews were completed between July 2021–March 2022.

### Survey

The online survey questions were modified from existing validated questionnaires or were newly created for this study by the authors. Questions were selected to address the study aims. Questions were modified from questionnaires designed to: (1) assess at risk pregnant women on a national level (PRIMS – Pregnancy Risk Assessment Monitoring System)^59^, (2) investigate the use of pregnancy apps^18^, and (3) assess body perception attitudes^60,61^. The authors assessed and tested the survey for face and content validity to ensure the questions captured the study aims. Several external researchers were also consulted. There were several rounds of feedback before the survey was finalised. The self-administered survey contained 49 questions and took approximately 20–30 min to complete. The majority of the survey questions were closed-ended (84%, 41/49) including multiple choice, dropdown, binary, ranking, and Likert questions. Questions were related to respondents’ health and health experiences during pregnancy, their health monitoring behaviour, their attitudes and usage of digital health tools, and thoughts about features included in the proposed app. At the end of the survey, respondents were asked two open-ended questions related to how the COVID-19 pandemic impacted their pregnancy and their use of digital health. Refer to Supplementary Method 1 for the survey in its entirety.

### Interviews

Participants who expressed their interest to participate in an interview at the end of the survey or who signed up for an interview via the advertising link, were asked to complete an online form assessing their eligibility and to provide their consent online. They were then contacted and instructed to schedule an interview via Calendly at a suitable time for them. Seventy percent (15/22) of the participants who provided their consent, scheduled an interview via Calendly. All interviews took place online, via Zoom video communications software, and were conducted by the same interviewer (NL) to ensure consistency. The interviewer was a female PhD student trained to conduct the interviews by a qualitative methodologist. Interviews lasted between 30–70 min (mean = 45 min, range = 33–70 min) and were semi-structured to allow for open discussion and elaboration of particular responses.

To ensure the quality of the online interviews, the following measures were taken: (i) all online interviews were conducted with video and audio, to capture both verbal and non-verbal cues; (ii) to build initial rapport, the interviewer would introduce themselves, the study, why it is being conducted, outline what to expect and address any questions/concerns; (iii) the interviewer reminded the participant that there are no right or wrong answers and that they may also refuse to answer any questions that they did not wish to during the interview; (iv) the interviewer also started the interview with a general question such as, “Is this a good time for you to talk?” or “Could you tell me a little about your pregnancy?”; (v) follow-up questions were asked when contextual information would add value to the conversation but was missing, unclear, not detailed. The interviewer also took extra care in reading non-verbal cues and included them in the interview transcripts in brackets when assessing them for accuracy against the video and audio recording. Additionally, the interviewer was aware of the circumstances and the subject of the interviews, and for this reason referred to the principles of trauma-informed research when interacting with the participants^62^.

Interview questions were created by the authors or modified from questions designed to assess weight related attitudes^22^. Qualitative interview topics were identified by reviewing the overall aims of the study and the survey questions. The interview was designed to address the study aims in a more in-depth manner: to investigate how digital health usage, self-monitoring behaviours and body image/weight may influence participants willingness to use digital health and their thoughts about the theoretical app features. The interview guide was organised in the following manner: (1) health monitoring and digital health usage; (2) weight and body image during pregnancy and weighing and photo taking behaviours; (3) theoretical app features; and (4) digital health usage during pregnancy in general. No theoretical framework was used to develop the interview guide. Refer to Supplementary Method 2 for the interview guide in its entirety.

### Data analyses

Survey respondents who completed 75% or more of the survey questions were included in the analysis. Multiple logistic regression was used to assess which variable predicted the use of pregnancy apps by survey participants. More specifically, the relationship between (i) BMI, (ii) age, (iii) gravidity, (iv) their usage of digital health more during pregnancy, (v) their belief that they can monitor themselves from home, (vi) any reported health condition and (vii) if they had concerns about data privacy and security issues in pregnancy apps were the predictor variables, and the ‘use of pregnancy apps’ was the response variable. Predictor variables were selected based on their theoretical and conceptual relevance. Only full survey responses were included in the logistic regression analysis. These results are reported as 95% confidence intervals and adjusted odds ratios. Open-source code published by “StatQuest with Josh Starmer”^63^ was used to run the logistic regressions. All statistical analyses were completed in R Studio version 4.2.0 (see Supplementary Method 3).

Participants reported relevant demographic information during interviews (whether they were currently pregnant, their location and gravidity). And participants education level and health status were recorded if participants chose to mention them during interviews. Interviews were recorded on Zoom and a digital voice recorder as a backup and the audio was transcribed automatically using Otter.ai software. The transcripts were proofread and imported into NVivo 12 for coding and analysis. The coding structure was first defined via an iterative process. Three authors (CP, CC, and NL) coded one initial interview and discussed the coding structure. Two authors (CP and NL) then coded two different interviews and finalised the coding structure. Coding between authors of these interviews was found to be highly consistent. One author coded all 15 interviews (NL) while another author coded 9 interviews (CP). Fig. 1 illustrates the major themes and subthemes that arose from the interviews. Once the interviews were coded, a coding comparison query was run in NVivo to numerically assess percentage agreement between coders (percentage of interview transcripts that should be coded to a specific node or case) and was found to be ≥93%. This confirmed that the coding was consistent. Common themes and subthemes from qualitative data in both the surveys and interviews were identified using a thematic analysis as described by Braun and Clarke^64^. The minimum sample size for interviews was determined to be 15 based on previous studies^65,66^. After interviewing, coding, and analysing 15 interviews, the research team (CP, NL, and CC) determined that data saturation was achieved. No new themes could be attained, therefore achieving inductive thematic saturation and data saturation as outlined by Saunders et al. (2018)^67^. All authors then discussed and confirmed the themes and subthemes based on the coding structure and study aims. All Figures were created using a Canva Pro Premium account.

### Reporting summary

Further information on research design is available in the Nature Research Reporting Summary linked to this article.

## Supplementary information

Supplement information

REPORTING SUMMARY
