# Supplementary material for: A mixed-methods study exploring women’s perceptions and recommendations for a pregnancy app with monitoring tools
Source: NPJ Digit Med. 2023 Mar 24;6:50. doi: 10.1038/s41746-023-00792-0 (PMC10036977; doi:10.1038/s41746-023-00792-0)
Supplement: Supplementary file 1 — Supplement information [file 41746_2023_792_MOESM1_ESM.pdf]

Supplementary Table 1. The demographics of the total of 108 survey respondents are presented.

Survey participant demographics (n 108)		
English as first language	n	%
Yes	89	82.4
No	19	17.6
Ancestry (regions)	n	%
Europe	68	63.0
N/A	16	14.8
Asia	12	11.1
Africa	5	4.6
Oceania	3	2.8
America	2	1.9
West Asia	2	1.9
Aboriginal or Torres Strait Islander	n	%
No	101	93.5
Aboriginal	3	2.8
Torres Strait Islander	0	0
I prefer not to say	4	3.7
Religion	n	%
Agnostic	14	13.0
Atheist	24	22.2
Chinese Traditional	1	0.9
Christianity	30	27.8
Hinduism	3	2.8
Islam	4	3.7
Non-religious	19	17.6
Rastafarianism	1	0.9
Other	5	4.6
I prefer not to say	7	6.5
Occupation	n	%
Arts & Entertainment	6	5.6
Business and finance	16	14.8
Healthcare and medicine	28	25.9
Not applicable	12	11.1
Other	22	20.4
Science and technology	10	9.3
Service occupation	14	13.0
Education	n	%
Secondary education or high school	18	16.7
General Education Development (GED)	3	2.8
Bachelor's degree	41	38.0
Master's degree	23	21.3
Doctorate or higher	13	12.0
Vocational qualification	9	8.3
No formal education	1	0.9
Marial Status	n	%
Married or de facto (living together)	102	94.4
Separated/divorced	1	0.9
Single	5	4.6
Widowed	0	0

Supplementary Table 2. The demographics of 15 interview participants are presented.

Interview participant demographics (n 15)		
Region participants were located	n	%
Australia	10	66.7
North America	3	20.0
Europe	1	6.7
Asia	1	6.7
	Participants who mentioned their education level and/or health status if it was relevant to their views/experiences	
Education level		
Completed tertiary education	8	53.3
Did not mention education	7	46.7
Developed health condition before, during or after pregnancy		
Developed health condition	9	60.0
Did not mention a developing a health condition	6	20.0

Supplementary Table 3. Table summarising the apps used by both survey participants and interview participants.

Pregnancy app usage	
Survey participants	%
What to Expect	28
Pregnancy+	26
Ovia Pregnancy	24
Flo	18
Baby Centre App	10
Sprout Pregnancy	3
Period Tracker	3
Interview participants	n
Ovia Pregnancy	3
Pregnancy+	2
What to Expect	2
Peanut	2
Pregnancy, Birth and Baby	1
Sprout Pregnancy	1
Clue Period Tracker	1
The Maternal and Child Health (MCH)	1

Supplementary Method 1

The study aims to facilitate and improve the health monitoring of women during pregnancy.

Your participation in this research study will help determine what ways we can design a pregnancy app that can improve how pregnant women monitor their health and communicate with their healthcare providers.

This study is being conducted by:

Dr Corinne Caillaud, Associate Professor, The University of SydneyMs. Nataša Lazarević, PhD student, The University of SydneyDr Céline Boehm, Professor, The University of SydneyDr Kathryn Williams, Senior Lecturer, Nepean Clinical School, University of Sydney & the Department of Endocrinology (Obesity), Nepean HospitalDr Gillian Rosic, Senior Dietician, Nepean Blue Mountains Family Metabolic Health Service, Nepean Clinical SchoolWe would also like to acknowledge Dr Alberto Krone Martins, Lecturer, Donald Bren School, School of Information and Computer Sciences, University of California, Irvine for his contribution.We estimate that the survey will take approximately 20-30 minutes to complete.

Participation in this study is voluntary. If you no longer wish to take part, please stop participating in the survey and close your browser.

At the end of the survey, you will have the opportunity to provide the researchers with your contact details if you would like to participate in the interview portion of this study or would like to receive further information.

Please read the Participant Information Sheet before you commence the survey. If you have any questions, please contact Nataša Lazarević: natasa.lazarevic@sydney.edu.au

I confirm that I have read the Participant Information Sheet in its entirety and understand its contents.	○ Yes ○ No
I understand that participation in this study is completely voluntary and that I am free to withdraw at any point, without reason or excuse and that withdrawal will result in the destruction of my existing data.	○ Yes ○ No
I consent to completing this survey.	○ Yes ○ No
I am pregnant	○ Yes ○ No

If you answered 'no' to any of these questions, please stop the survey now because your responses will not be included in the study.
Thank you for your time.

This survey includes different sections. In this first section we will ask you questions about you. In the later sections we will ask you questions about your willingness to use technology during pregnancy.

Country of residence

◯ Afghanistan ◯ Albania ◯ Algeria ◯ Andorra ◯ Angola ◯ Antigua & Deps ◯ Argentina
◯ Armenia ◯ Australia ◯ Austria ◯ Azerbaijan ◯ Bahamas ◯ Bahrain ◯ Bangladesh
◯ Barbados ◯ Belarus ◯ Belgium ◯ Belize ◯ Benin ◯ Bhutan ◯ Bolivia ◯ Bosnia
Herzegovina ◯ Botswana ◯ Brazil ◯ Brunei ◯ Bulgaria ◯ Burkina ◯ Burundi
◯ Cambodia ◯ Cameroon ◯ Canada ◯ Cape Verde ◯ Central African Rep ◯ Chad
◯ Chile ◯ China ◯ Colombia ◯ Comoros ◯ Congo ◯ Congo {Democratic Rep}
◯ Costa Rica ◯ Croatia ◯ Cuba ◯ Cyprus ◯ Czech Republic ◯ Denmark ◯ Djibouti
◯ Dominica ◯ Dominican Republic ◯ East Timor ◯ Ecuador ◯ Egypt ◯ El Salvador
◯ Equatorial Guinea ◯ Eritrea ◯ Estonia ◯ Ethiopia ◯ Fiji ◯ Finland ◯ France
◯ Gabon ◯ Gambia ◯ Georgia ◯ Germany ◯ Ghana ◯ Greece ◯ Grenada
◯ Guatemala ◯ Guinea ◯ Guinea-Bissau ◯ Guyana ◯ Haiti ◯ Honduras ◯ Hungary
◯ Iceland ◯ India ◯ Indonesia ◯ Iran ◯ Iraq ◯ Ireland {Republic} ◯ Israel
◯ Italy ◯ Ivory Coast ◯ Jamaica ◯ Japan ◯ Jordan ◯ Kazakhstan ◯ Kenya
◯ Kiribati ◯ Korea North ◯ Korea South ◯ Kosovo ◯ Kuwait ◯ Kyrgyzstan ◯ Laos
◯ Latvia ◯ Lebanon ◯ Lesotho ◯ Liberia ◯ Libya ◯ Liechtenstein ◯ Lithuania
◯ Luxembourg ◯ Macedonia ◯ Madagascar ◯ Malawi ◯ Malaysia ◯ Maldives
◯ Mali ◯ Malta ◯ Marshall Islands ◯ Mauritania ◯ Mauritius ◯ Mexico ◯ Micronesia
◯ Moldova ◯ Monaco ◯ Mongolia ◯ Montenegro ◯ Morocco ◯ Mozambique
◯ {Burma} ◯ Namibia ◯ Nauru ◯ Nepal ◯ Netherlands ◯ New Zealand ◯ Nicaragua
◯ Niger ◯ Nigeria ◯ Norway ◯ Oman ◯ Pakistan ◯ Palau ◯ Panama ◯ Papua New Guinea
◯ Paraguay ◯ Peru ◯ Philippines ◯ Poland ◯ Portugal ◯ Qatar ◯ Romania
◯ Russian Federation ◯ Rwanda ◯ St Kitts & Nevis ◯ St Lucia ◯ Saint Vincent & the Grenadines
◯ Samoa ◯ San Marino ◯ Sao Tome & Principe ◯ Saudi Arabia ◯ Senegal ◯ Serbia
◯ Seychelles ◯ Sierra Leone ◯ Singapore ◯ Slovakia ◯ Slovenia ◯ Solomon Islands
◯ Somalia ◯ South Africa ◯ South Sudan ◯ Spain ◯ Sri Lanka ◯ Sudan ◯ Suriname
◯ Swaziland ◯ Sweden ◯ Switzerland ◯ Syria ◯ Taiwan ◯ Tajikistan ◯ Tanzania
◯ Thailand ◯ Togo ◯ Tonga ◯ Trinidad & Tobago ◯ Tunisia ◯ Turkey ◯ Turkmenistan
◯ Tuvalu ◯ Uganda ◯ Ukraine ◯ United Arab Emirates ◯ United Kingdom ◯ United States
◯ Uruguay ◯ Uzbekistan ◯ Vanuatu ◯ Vatican City ◯ Venezuela ◯ Vietnam
◯ Yemen ◯ Zambia ◯ Zimbabwe

Postcode

____
(Or area code/ZIP code if outside of Australia)

How long have you been residing at your address?

____
(Please specify years or months)

Is English your first language?

◯ Yes
◯ No

If no, please indicate what your first language is.

____

Are you of Aboriginal or Torres Strait Islander Origin?
((For persons of both Aboriginal and Torres Strait Islander origin, check both "Yes" boxes))

☐ No
☐ Yes, Aboriginal
☐ Yes, Torres Strait Islander
☐ I prefer not to say

What is your ancestry? If you prefer not to say type N/A.

____
((For example: English, Scottish, South African, Malaysian, Vietnamese. Provide more than one ancestry if necessary.))

What is your religion?
(For example: Judaism, Islam, Greek Orthodox, Buddhism and Baptist. Please choose an option from the dropdown list.)

○ I prefer not to say ○ African Traditional & Diasporic ○ Agnostic ○ Atheist ○ Baha'i ○ Buddhism ○ Cao Dai ○ Chinese traditional religion ○ Christianity ○ Hinduism ○ Islam ○ Jainism ○ Juche ○ Judaism ○ Neo-Paganism ○ Nonreligious ○ Rastafarianism ○ Secular ○ Shinto ○ Sikhism ○ Spiritism ○ Tenrikyo ○ Unitarian-Universalism ○ Zoroastrianism ○ primal-indigenous ○ Other

If other, please indicate what your religion is.

____

What describes your occupation best?

○ Arts and entertainment ○ Business and finance ○ Healthcare and medicine ○ Science and technology ○ Service occupation (Providing a service for people) ○ Not applicable ○ Other

If other, what is your occupation?

____

What is the highest level of school or degree that you have completed?

○ No formal education ○ Primary education ○ Secondary education or high school ○ General Education Development (GED) ○ Vocational qualification ○ Bachelor's degree ○ Master's degree ○ Doctorate or higher

Marital status

☐ Married or de facto (living together)
☐ Separated/Divorced
☐ Single
☐ Widowed

What is your year of birth?

____

How many people live with you in your house (not counting yourself)?

____

What week of gestation are you in?
(Weeks)

◯ 1 ◯ 2 ◯ 3 ◯ 4 ◯ 5 ◯ 6 ◯ 7 ◯ 8 ◯ 9 ◯ 10 ◯ 11 ◯ 12 ◯ 13
◯ 14 ◯ 15 ◯ 16 ◯ 17 ◯ 18 ◯ 19 ◯ 20 ◯ 21 ◯ 22 ◯ 23 ◯ 24 ◯ 25
◯ 26 ◯ 27 ◯ 28 ◯ 29 ◯ 30 ◯ 31 ◯ 32 ◯ 33 ◯ 34 ◯ 35 ◯ 36 ◯ 37
◯ 38 ◯ 39 ◯ 40 ◯ 40 +

Before your current pregnancy, how many children have you carried to full term?

◯ 0 ◯ 1 ◯ 2 ◯ 3 ◯ 4 ◯ 5 ◯ 6 ◯ 7 ◯ 8 ◯ 9 ◯ 10 ◯ 10+

Your survey progress:
26%

.

The next questions are about the time BEFORE you got pregnant with your new baby.

Just before you got pregnant with your new baby, how much did you weigh?

____

(Kilograms)

How tall are you without shoes?

____

(cm)

Your survey progress:
30%

.

During the 3 months before you got pregnant with your new baby, had you been told by a doctor that you had any of the following conditions?
For each one, check NO if you did not have the condition or YES if you did.

	No	Yes
Type 1 or Type 2 diabetes (not gestational diabetes or diabetes that starts during pregnancy)	◯	◯
High blood pressure or hypertension	◯	◯
Depression	◯	◯
Polycystic Ovarian Syndrome	◯	◯
Non-alcoholic fatty liver disease	◯	◯
Obstructive sleep apnoea	◯	◯
Other	◯	◯

If other, please state which condition you had been diagnosed with.

____

Thinking back to just before you got pregnant with your new baby, how did you feel about becoming pregnant? Select ONE answer

◯ I wanted to be pregnant later
◯ I wanted to be pregnant sooner
◯ I wanted to be pregnant then
◯ I didn't want to be pregnant then or at any time in the future
◯ I wasn't sure what I wanted

In the 12 months before you got pregnant with your new baby, did you have any health care visits with a doctor, nurse, or other health care worker, including a dental or mental health worker?

◯ Yes
◯ No

What type of health care visit/s did you have in the 12 months before you got pregnant with your new baby? Select ALL that apply.

☐ Regular checkup at my family general practitioners (GP's) office
☐ Regular checkup at my Obstetrician/Gynaecologist's (OB/GYN's) office
☐ Visit for an illness or chronic condition
☐ Visit for an injury
☐ Visit for family planning or birth control
☐ Visit for depression or anxiety
☐ Visit to have my teeth cleaned by a dentist
☐ Other

If other, please state what kind of health care visit did you have.

____

Your survey progress:
37%

.

The next questions are related to how you monitor your body and some other aspects of your health DURING pregnancy.

How often do you weigh yourself?

- ◯ Daily
- ◯ 5 to 7 times a week
- ◯ 2 to 4 times a week
- ◯ Once weekly
- ◯ 2 to 3 times a month
- ◯ Monthly
- ◯ A few times a year
- ◯ I do not weigh myself

Do you agree or disagree with the following statement:

I feel that I can monitor my pregnancy easily from home.

- ◯ Strongly agree
- ◯ Agree
- ◯ Do not disagree or agree
- ◯ Disagree
- ◯ Strongly disagree

Do you use any activity trackers such as fitbits or smart watches during pregnancy?
Select all the options that apply.

- ☐ Apple/Samsung/Fossil smartwatch
- ☐ Fitbits like the Inspire, Charge and Versa models
- ☐ Garmin fitness trackers like Vivosmart and Forerunner
- ☐ I do not use activity trackers
- ☐ Others

If other, please state what activity trackers you use.

____

If you use activity trackers, how often do you achieve your exercise goals?

- ☐ Daily
- ☐ 5 to 7 times a week
- ☐ 2 to 4 times a week
- ☐ Once weekly
- ☐ I do not really try to meet the objectives set by the app

---

Do you agree or disagree with the following statement:

You have ACCESS to all the information you need to make informed decisions about physical activity and exercise during pregnancy.

- ◯ Strongly agree
- ◯ Agree
- ◯ Do not disagree or agree
- ◯ Disagree
- ◯ Strongly disagree

Do you use any app to monitor your diet during pregnancy?

◯ Yes
◯ No

If yes, please indicate what diet app you use.

____

How often do you achieve dietary objectives?

◯ Daily
◯ 5 to 7 times a week
◯ 2 to 4 times a week
◯ Once weekly
◯ Rarely
◯ I do not really try to meet the objectives set by the app

Your survey progress:
50%

.

The next questions are about your feelings about the changes that happen due to pregnancy.

Do you agree or disagree with the following statement:

I worry about putting on too much weight during pregnancy

◯ Strongly agree
◯ Agree
◯ Do not disagree or agree
◯ Disagree
◯ Strongly disagree

Being pregnant modified my physical activity or participation in structured exercise sessions?

◯ Yes
◯ No

Do you agree or disagree with the following statement:

I feel good and energised since the start of my pregnancy

◯ Strongly agree
◯ Agree
◯ Do not disagree or agree
◯ Disagree
◯ Strongly disagree

Your survey progress:
56%

.

Please RANK the following parts of your body in order of how happy/satisfied you feel about them during pregnancy. 1 being the most important and 6 being the least important.

***Each body region must be included in your ranking.**
***No two body regions can have the same rank.**

	1	2	3	4	5	6
Your chest	◯	◯	◯	◯	◯	◯
The size/width of your shoulders	◯	◯	◯	◯	◯	◯
The size/width of your waist and hip	◯	◯	◯	◯	◯	◯
Your ankles	◯	◯	◯	◯	◯	◯
Your arms	◯	◯	◯	◯	◯	◯
Your hands	◯	◯	◯	◯	◯	◯

Your survey progress:
57%

.

The next questions are about your attitudes and usage of digital tools during pregnancy.

Have you noticed that you use apps and search the internet more now that you are pregnant?

◯ Yes
◯ No

During your pregnancy, what online resources do you PREFER to use to access pregnancy-related information? Select all that apply.

☐ Websites (i.e. blogs, medical information websites)
☐ Social networking sites (i.e., Instagram, Facebook, Twitter, etc..)
☐ YouTube videos
☐ Podcasts
☐ Using a pregnancy app
☐ e-Books
☐ I do not use online resources for pregnancy information
☐ Other

If other, please indicate what resource you use to access pregnancy-related information.

____

Which social networking sites do you PREFER to use to access pregnancy-related information? Select all that apply.

☐ Twitter
☐ Facebook
☐ Instagram
☐ LinkedIn
☐ Youtube
☐ Whatsapp
☐ Messenger
☐ WeChat
☐ Tumblr
☐ Tik Tok
☐ Weibo
☐ Reddit
☐ Viber
☐ Snapchat
☐ Pinterest
☐ Telegram
☐ I do not use social networking for pregnancy information
☐ Other

If other, please indicate what social media networks you use to access pregnancy-related information.

____

Which online/digital source of information do you TRUST most for information on pregnancy? Select ALL that apply.

☐ Websites (i.e. blogs, medical information websites)
☐ social networking sites (i.e., Instagram, Facebook, Twitter, etc..)
☐ YouTube videos
☐ Podcasts
☐ Using a pregnancy app
☐ e-Books
☐ Other

If other, please state which online/digital source of information you TRUST most.

____

During your pregnancy, what other resources do you feel comfortable to use to access pregnancy-related information? Select all that apply.

☐ Asking a health care worker
☐ Asking friends
☐ Asking a family member
☐ Reading books
☐ Leaflets
☐ Other

If other, please state what other resources you use to access pregnancy-related information.

____

Which pregnancy app do you prefer to use to access pregnancy-related information?
Type "N/A" if you do not use any.

For what purposes do you use pregnancy apps? Select all options that apply.

☐ As a source of information
☐ For education
☐ For self-monitoring
☐ For reassurance that the pregnancy is going well
☐ Other
☐ I do not use pregnancy apps

If other, please indicate what you use pregnancy apps for.

____

What did you find most useful or helpful about these apps?

Are you concerned by the services offered by the app?

◯ Yes
◯ No
◯ N/A

Are you concerned about data privacy and security issues in relation to using the apps?

◯ Yes
◯ No
◯ N/A

Your survey progress:
76%

.

The next questions are related to your attitudes to a new app we are designing.

From a photo/image taken on a smartphone by you, the app will be able to calculate changes in your body dimensions and shape over time. Algorithms will make these calculations and the images will be saved on a secured server and will NOT be available to anyone.

How often do you take selfies?

◯ Never
◯ Seldom
◯ Sometimes
◯ Often
◯ Always

How often do you take photographs of your entire body?

◯ Never
◯ Seldom
◯ Sometimes
◯ Often
◯ Always

How often do you take family photos?

◯ Never
◯ Seldom
◯ Sometimes
◯ Often
◯ Always.

Would you be comfortable taking photos of your entire body regularly so the app can calculate changes in your body dimensions?

◯ Yes
◯ No
◯ I don't know

Would you be comfortable taking photos of specific body parts regularly so the app can calculate changes in your body dimensions?

◯ Yes
◯ No
◯ I don't know

If your answer is NO, why?

____

The app will also be able to collect other health information important for your pregnancy (diet, physical activity, smoking habits etc...).

Which information would you be comfortable adding to the app during your pregnancy? You can make multiple choices.

☐ Physical activity
☐ Blood pressure
☐ Blood glucose
☐ Smoking habits
☐ Alcohol consumption
☐ Mood
☐ Diet
☐ Body temperature
☐ Not interested
☐ Other

If other, please state which health information you would be comfortable adding to the app.

____

Your survey progress:
87%

.

In the app you will have the option to share the health results (not the pictures) generated from the app and the health information added by you with your chosen healthcare professional (e.g. your clinician, midwife or obstetrician). The healthcare professionals will then be able to provide individualised advice to you.

Would you be comfortable sharing the health results generated from the app (e.g. body measurement results) with your health professional (e.g. your clinician, midwife or obstetrician) through a secure network?

◯ Yes
◯ No
◯ I am not sure

The app technology will be able to learn from anonymous data from pregnant women and healthcare professionals and will be able to assist in the identification, prediction and prevention of adverse health outcomes during pregnancy.

How interested are you to use such an app?

◯ Extremely interested
◯ Very interested
◯ Moderately interested
◯ Slightly interested
◯ Not at all interested

Would you be comfortable with your anonymised data being used to train and develop the app technology while using the app?

◯ Yes
◯ No
◯ I am not sure

If you could create any pregnancy app or tool what features would it include? Select all that apply.

☐ Provide you with information about nutrition
☐ Provide you with information about physical activity and exercise
☐ Provide you with information about gestational weight gain
☐ Provide you with personalised feedback and advice
☐ Connect you with a community of other mothers
☐ Allow you to monitor the development of your baby
☐ Allow you to monitor your body
☐ Other

If other, please state which features would you want to include in a pregnancy app.

____

How has the COVID-19 pandemic and the government's response impacted your opinions about the use of digital health and pregnancy apps?
Type "N/A" if you have not been impacted.

How has the social distancing, self-isolation and quarantine measures impacted the course of your pregnancy?
Type "N/A" if you have not been impacted.

Your survey progress:
100% complete, please submit the survey

.

Supplementary Method 2

Introduction

- Introduction of researcher and overview of the study.
- Acknowledge that the interview will be confidential.

Body-related questions

1. **How are you feeling? Could you tell me a little about your pregnancy?**

2. **Did you experience any barriers preventing you from monitoring your health during pregnancy?**
    - Example of barriers: cost of care is expensive, the pandemic no or inadequate insurance plan, long commute to nearest health facility, delays in receiving the appropriate care, and family situation (using a show card with examples printed in large font)

Potential follow-up – How did the pandemic influence how frequently you had medical appointments?

3. **What is your experience of using apps or websites to self-monitor your health** (like for keeping track of your diet, physical activity, or weight)?

4. **Did you also seek and use web-based information to make decisions related to your health during pregnancy?** In general, which health apps do you use if any?

5. **How did you feel about your body weight/image during pregnancy compared to before you were pregnant? And now?** Potential follow-up – Did you notice any changes in your relationship with your body?

6. **Other than your belly region, in what regions of your body did you notice the most changes during of pregnancy? And what kind of change?** Potential follow-up – Where did you notice the most change in shape and size of your body?

7. **Did you feel that anxiety and stress influenced your monitoring behaviour? Potential** follow-up – Like how frequently you weighed yourself? How did it influence it?

8. **Did you monitor your weight and/or body shape regularly? (Yes or no response) How did you do this? How often?**

9. **How did you feel when you weighed yourself?** Potential follow-up – Do feel happy, comfortable, uncomfortable etc.

10. **How often did you take photos of yourself? How did you feel when you took photos of yourself/selfies?**

We will now ask you questions about a theoretical app:

11. **During pregnancy would you have been comfortable taking photos of your body or body parts regularly in an app that monitors how your body is growing and changing and would be able to also give you feedback about your health?**

12. **Would you have been comfortable communicating with and sharing your health information with your chosen healthcare professional via an app?** Potential follow-up: What about if that health information was body measurements?

13. **How do you think communicating via an app with your healthcare professional (such as your clinician, obstetrician-gynaecologist (OBGYN), midwife and dietician) would have impacted your pregnancy care?**

14. **In your opinion do you think the use of technology would have improved the healthcare you received during pregnancy? Could it have made your healthcare more personalized?**

15. **Did you feel like you had enough information about your pregnancy? Did you feel supported?**

16. **In what way did you think the healthcare provided during your pregnancy could have been better tailored to you and your needs?** Potential follow-up: did you find that the information you received was specific or general?

17. **Are there any other comments you want to raise regarding the use of digital tools to monitor pregnancy?**

18. **Did the COVID-19 pandemic impact how willing you were to access health information digitally and use digital tools like pregnancy apps?** Potential follow-up: How has the pandemic influence how trusting you are of the health information?

Supplementary Method 3

```r
## logistic Regression tutorial by https://www.youtube.com/watch?v=C4N3_XJJ-jU
# Code found here https://github.com/StatQuest/logistic_regression_demo/blob/master/logistic_regression_demo.R

install.packages("car")
install.packages('cowplot')

library(ggplot2)
library(cowplot)
library(stats)
library(readxl)
library(REdaS)


###############read in the data set
###############DHU = Digital Health Usage

DHU <- read_excel("~/Desktop/PhD/Study1/DigitalHealthUsage_LR.xlsx")
View(DHU)
attach(DHU)
head(DHU, 3)

##
## Reformat the data so that it is
## 1) Easy to use (add nice column names)
## 2) Interpreted correctly by glm()..

str(DHU) # this shows that we need to tell R which columns contain factors
# it also shows us that there are some missing values. There are "?"s
# in the dataset. These are in the "ca" and "thal" columns...

## Now add factors for variables that are factors and clean up the factors
## that had missing data...
#DHU[DHU$sex == 0,]$sex <- "F"
#data[data$sex == 1,]$sex <- "M"
DHU$BMI <- as.factor(DHU$BMI)
DHU$WeightWorry <- as.factor(DHU$WeightWorry)
DHU$ReportedHealthCondition <- as.factor(DHU$ReportedHealthCondition)
DHU$VisitHCP <- as.factor(DHU$VisitHCP)
DHU$DataPrivacyConcerns <- as.factor(DHU$DataPrivacyConcerns)
DHU$SelfmonitoringAtHome <- as.factor(DHU$SelfmonitoringAtHome)
DHU$UsePregApps <- as.factor(DHU$UsePregApps)


logistic <- glm(UsePregApps ~ BMI + Age + WeightWorry + ReportedHealthCondition
                + GestationWeek + VisitHCP + DataPrivacyConcerns + SelfmonitoringAtHome, data=DHU, family="binomial")
summary(logistic)


## Now calculate the overall "Pseudo R-squared" and its p-value

## NOTE: Since we are doing logistic regression...
## Null devaince = 2*(0 - LogLikelihood(null model))
##               = -2*LogLikihood(null model)
## Residual deviacne = 2*(0 - LogLikelihood(proposed model))
##                   = -2*LogLikelihood(proposed model)

ll.null <- logistic$null.deviance/-2
ll.proposed <- logistic$deviance/-2

## McFadden's Pseudo R^2 = [ LL(Null) - LL(Proposed) ] / LL(Null)
(ll.null - ll.proposed) / ll.null
```

```
## chi-square value = 2*(LL(Proposed) - LL(Null))
## p-value = 1 - pchisq(chi-square value, df = 2-1)

chisq <- 1 - pchisq(2*(ll.proposed - ll.null), df=(length(logistic$coefficients)-1))

#Running confidence intervals

confint(logistic) #95% CI for the coefficients

#Odds ratios
exp(coef(logistic))

#drive the output data to txt file
sink('PregAppUsageLR.txt')
summary(logistic)
sink()

#terminates the connection
unlink('PregAppUsageLR.txt')
```
