# Supplementary material for: A mixed-methods study exploring women’s perceptions and recommendations for a pregnancy app with monitoring tools
Source: NPJ Digit Med. 2023 Mar 24;6:50. doi: 10.1038/s41746-023-00792-0 (PMC10036977; doi:10.1038/s41746-023-00792-0)
Supplement: Supplementary file 2 — REPORTING SUMMARY [file 41746_2023_792_MOESM2_ESM.pdf]

Corresponding author(s): Prof Corinne Caillaud and Natasa Lazarevic

Last updated by author(s): 2022-12-6

Reporting Summary

Nature Portfolio wishes to improve the reproducibility of the work that we publish. This form provides structure for consistency and transparency in reporting. For further information on Nature Portfolio policies, see our Editorial Policies and the Editorial Policy Checklist.

Statistics

For all statistical analyses, confirm that the following items are present in the figure legend, table legend, main text, or Methods section.

n/a	Confirmed	
☐	☒	The exact sample size (*n*) for each experimental group/condition, given as a discrete number and unit of measurement
☒	☐	A statement on whether measurements were taken from distinct samples or whether the same sample was measured repeatedly
☒	☐	The statistical test(s) used AND whether they are one- or two-sided<br>*Only common tests should be described solely by name; describe more complex techniques in the Methods section.*
☐	☒	A description of all covariates tested
☒	☐	A description of any assumptions or corrections, such as tests of normality and adjustment for multiple comparisons
☐	☒	A full description of the statistical parameters including central tendency (e.g. means) or other basic estimates (e.g. regression coefficient) AND variation (e.g. standard deviation) or associated estimates of uncertainty (e.g. confidence intervals)
☒	☐	For null hypothesis testing, the test statistic (e.g. *F*, *t*, *r*) with confidence intervals, effect sizes, degrees of freedom and *P* value noted<br>*Give P values as exact values whenever suitable.*
☒	☐	For Bayesian analysis, information on the choice of priors and Markov chain Monte Carlo settings
☒	☐	For hierarchical and complex designs, identification of the appropriate level for tests and full reporting of outcomes
☒	☐	Estimates of effect sizes (e.g. Cohen's *d*, Pearson's *r*), indicating how they were calculated

Our web collection on statistics for biologists contains articles on many of the points above.

Software and code

Policy information about availability of computer code

Data collection	REDCap to collect survey data, Calendly to schedule the interviews, Zoom to record the audio and video of interviews, Otter.ai to create the interview transcripts.
Data analysis	NVivo 12 was used to analyse the interviews. All statistical analyses were completed in R Studio version 4.2.0 using this open source code to run the logistic regression: https://www.youtube.com/watch?v=C4N3_XJJ-jU

For manuscripts utilizing custom algorithms or software that are central to the research but not yet described in published literature, software must be made available to editors and reviewers. We strongly encourage code deposition in a community repository (e.g. GitHub). See the Nature Portfolio guidelines for submitting code & software for further information.

Data

Policy information about availability of data

All manuscripts must include a data availability statement. This statement should provide the following information, where applicable:

- Accession codes, unique identifiers, or web links for publicly available datasets
- A description of any restrictions on data availability
- For clinical datasets or third party data, please ensure that the statement adheres to our policy

All data available upon reasonable request to corresponding author.

Human research participants

Policy information about studies involving human research participants and Sex and Gender in Research.

Reporting on sex and gender	Sex and gender is not reported. All participants were pregnant and/or recently pregnant.
Population characteristics	Population characteristics that were reported include: location, age, trimester, gravidity, education, and health status.
Recruitment	Survey and interview participants were recruited online and in-person. In person recruitment Participants were recruited both in person and online. In person recruitment was conducted at the Nepean Hospital Antenatal Clinic, Australia. Online recruitment was conducted via social media posts and advertisements as well as via email newsletter advertisements. A potential self-selection bias could have been participants more interested in digital health and apps were more likely to participate.
Ethics oversight	Ethics approval was obtained from Nepean Blue Mountains Local Health District Human Research Ethics Committee, Australia (Ethics approval number: ETH00580). Online consent was obtained from all participants.

Note that full information on the approval of the study protocol must also be provided in the manuscript.

Field-specific reporting

Please select the one below that is the best fit for your research. If you are not sure, read the appropriate sections before making your selection.

☐ Life sciences ☒ Behavioural & social sciences ☐ Ecological, evolutionary & environmental sciences

For a reference copy of the document with all sections, see nature.com/documents/nr-reporting-summary-flat.pdf

Behavioural & social sciences study design

All studies must disclose on these points even when the disclosure is negative.

Study description	A convergent mixed method approach was used. Quantitative and qualitative data were collected concurrently and analysed separately through surveys and interviews. The participants could complete both the survey and interview, but their data were not linked. The study findings and interpretations were triangulated from the combined data.
Research sample	108 survey participants (101 completed and 7 partial responses) who were pregnant at the time of survey completion: Three quarters of the participants (76%) were in Australia. The majority of the participants were between the ages of 18 and 35 (71%), were in their second or third trimesters (84%), were experiencing their first pregnancy (55%), were in the normal and overweight BMI ranges (71%), completed tertiary education (71%), and reported no health conditions (65%).<br>The 15 interview participants were either currently pregnant or recently pregnant in the last 12 months: The majority of the interview participants were recently pregnant and in their postnatal period (10/15, 67%), while the rest were pregnant at the time of the interview. Fifty-three percent (8/15) reported that it was their first pregnancy. Majority of participants were in Australia (10/15).<br>The samples are not fully representative across all demographics such as country location, BMI category and socioeconomic status and this is listed as a limitation in our manuscript. Given that this study is exploratory, the collection of responses offers an insight into the views and recommendations of pregnant women.
Sampling strategy	Quantitative data (surveys): A random sampling procedure was chosen. This method was chosen to increase the generalizability of the results. The procedure, however, meant that we could not control for representation across all demographics as done using purposive sampling. Given that the study is exploratory, and recruitment occurred during the pandemic, participants who wanted to share within the timing outlined below were included in the study.<br><br>Qualitative data (interviews): Common themes and subthemes from qualitative data in both the surveys and interviews were identified using a thematic analysis. The research team concluded that data saturation for the interviews was met after the analysis was completed. Analysis was completed in an iterative manner and a coding structure was outlined based on the study aims. All authors then discussed and confirmed the themes and subthemes based on the coding structure and study aims. Data saturation was determined by referring to the coding structure and aims, and authors felt that enough data had been collected and there were no new insights being drawn.
Data collection	Surveys online and self-administered. The software used to collect the data was REDCap.<br>Interviews were recorded on Zoom and a digital voice recorder as a backup and the audio was transcribed automatically using Otter.ai software. The transcripts were proofread and imported into NVivo 12 for coding and analysis. Only the researcher and participant were present during the zoom interview.
Timing	Survey recruitment occurred during November 2020 – May 2022 and the interviews were completed between July 2021- March 2022.
Data exclusions	No data were excluded.

Non-participation	No participants dropped out/declined participation.
Randomization	Participants were not allocated to experimental groups.

Reporting for specific materials, systems and methods

We require information from authors about some types of materials, experimental systems and methods used in many studies. Here, indicate whether each material, system or method listed is relevant to your study. If you are not sure if a list item applies to your research, read the appropriate section before selecting a response.

Materials & experimental systems

n/a	Involved in the study	
☒	☐	Antibodies
☒	☐	Eukaryotic cell lines
☒	☐	Palaeontology and archaeology
☒	☐	Animals and other organisms
☒	☐	Clinical data
☒	☐	Dual use research of concern

Methods

n/a	Involved in the study	
☒	☐	ChIP-seq
☒	☐	Flow cytometry
☒	☐	MRI-based neuroimaging
